# Supplementary material for: PCNs, PCBs, and PCDD/Fs in Soil around a Cement Kiln Co-Processing Municipal Wastes in Northwestern China: Levels, Distribution, and Potential Human Health Risks
Source: Int J Environ Res Public Health. 2022 Oct 7;19(19):12860. doi: 10.3390/ijerph191912860 (PMC9566703; doi:10.3390/ijerph191912860)
Supplement: Supplementary file 1 [file ijerph-19-12860-s001.zip › ijerph-1941227-supplementary.pdf]

Supplementary Material

PCNs, PCBs, and PCDD/Fs in soil around a cement kiln co-processing municipal wastes in Northwestern China: Levels, distribution, and potential human health risks

Jiali Han[a], Chenyang Xu[a], Jun Jin [a,b,c], Jicheng Hu [a,b,c]*

[a] *College of Life and Environmental Science, Minzu University of China, Beijing 100081, China*

[b] *Key Laboratory of Ecology and Environment in Minority Areas (Minzu University of China), National Ethnic Affairs Commission, Beijing 100081, China*

[c] *Beijing Engineering Research Center of Food Environment and Public Health, Minzu University of China, Beijing 100081, China*

*Corresponding author

Tel.: +86 10 68932242; Fax: +86 10 68932633; E-mail: jichhu@126.com

Text S1	The detailed information for the plant and study area.
Figure S1	Contributions of the PCDD/Fs, PCNs, and PCBs to the total PCNs, PCDD/Fs, and PCBs concentrations and TEQ values.
Table S1	Compounds of standard and internal standard mix solution
Table S2	Toxic equivalency factors (TEFs) of PCDD/Fs, PCBs, and PCNs
Table S3	Calculation parameters used in the health risk assessment
Table S4	Compound specific parameters used in the health risk assessment
Table S5	Pearson correlation coefficients for the relationships between the soil and fly ash samples of PCN, PCB and PCDD/Fs homologs
Table S6	Carcinogenic risks (CR) and non-carcinogenic risks (no-CR) near by the cement kilns co-processing municipal waste

Table S7	Proportions of carcinogenic risk (CR) and non-carcinogenic risks (no-CR) in different pathways (%)

Text S1

The following is the detailed information of the cement kilns co-processing municipal waste. The plant was established in 2015, using a new dry-process kiln to co-process municipal wastes, with a waste treatment capacity of about 110,000 t per year. The plant mainly uses limestone and clay as raw materials for cement clinkers production, with a 4500 t production capacity per day. About 6500 t raw materials are used to produce cement clinkers per day. The kilns is equipped with an electrostatic precipitator and the dust collected by the dust catcher was finally put back into cement production. The dust emission is approximately 80 t per year. The stack is 90 m high The plant is equipped with an online monitoring system to monitor pollutants, such as dust, nitrogen oxide, sulfur dioxide, and hydrogen chloride. Nitrogen oxide is the main pollutant in flue gas, abatement by non-catalytic reduction (SNCR), but still emits at a rate of 900 t per year.

In the study area, the mean annual precipitation is about 511 mm, and the average number of sunny days is 113 per year. The dominant wind direction in this area is west-northwest. However, we found no regular variation of concentrations with dominant wind direction. Perhaps because the cement kiln is located in a valley, with a mountain-valley wind circulation system. The mountain-valley wind system is composed of light-dark cycle of mountain and valley winds, which are caused by the temperature difference of air. The valley wind blows from the valley to the mountain slopes during the day, and the mountain wind blows from the mountain slopes to the valley at night, repeating the cycle [1]. Thus, mountain-valley wind has obvious influence on pollutant transport. Pollutants are removed with the valley wind and then enter with the mountain wind, repeatedly circulating, and there may be no dependence on dominant wind direction.

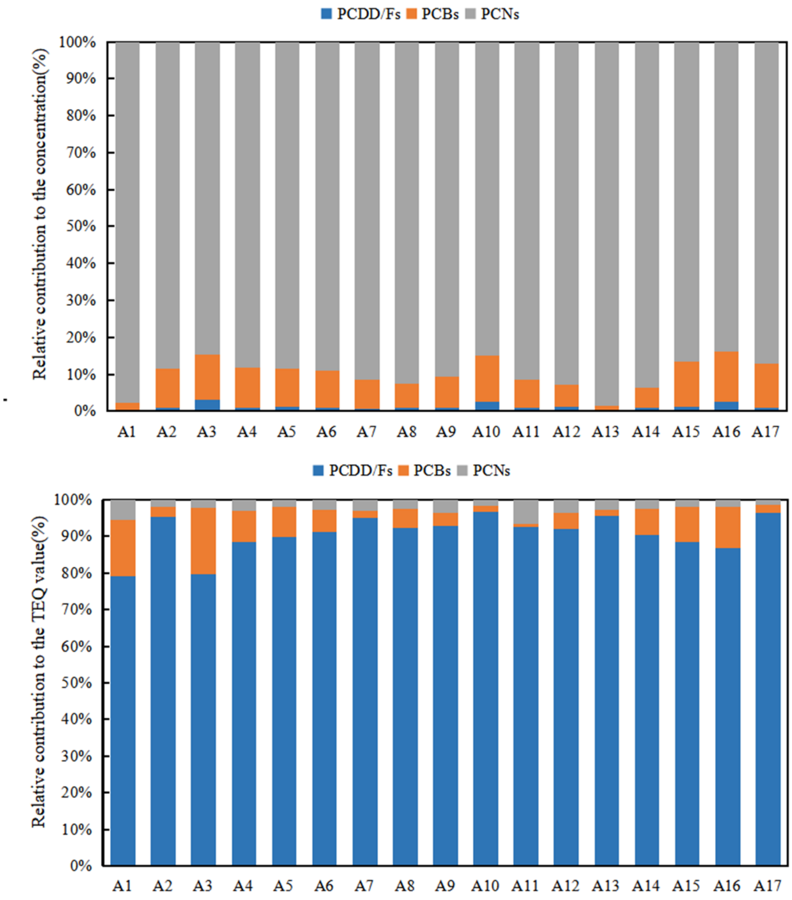

Figure S1. Contributions of the PCDD/Fs, PCNs, and PCBs to the total PCNs, PCDD/Fs, and PCBs concentrations and TEQ values.

Table S1. Compounds of standard and internal standard mix solution

1613 STOCK and DF-LCS-C ($^{13}C_{12}$-labeled)			
2,3,7,8-TCDF	2,3,7,8-TCDD	1,2,3,7,8-PeCDF	2,3,4,7,8-PeCDF
1,2,3,7,8-PeCDD	1,2,3,4,7,8-HxCDF	1,2,3,6,7,8-HxCDF	2,3,4,6,7,8-HxCDF
1,2,3,7,8,9-HxCDF	1,2,3,4,7,8-HxCDD	1,2,3,6,7,8-HxCDD	1,2,3,7,8,9-HxCDD
1,2,3,4,6,7,8-HpCDF	1,2,3,4,7,8,9-HpCDF	1,2,3,4,6,7,8-HpCDD	OCDF
OCDD			
PCBs internal standard ($^{13}C_{12}$-labeled)			
PCB-28	PCB-52	PCB-77	PCB-81
PCB-101	PCB-105	PCB-104	PCB-118
PCB-123	PCB-126	PCB-156	PCB-157
PCB-167	PCB-169	PCB-170	PCB-180
PCB-189			
ECN-5102 ($^{13}C_{10}$-labeled)			
1,2,3,4-TetraCN	1,3,5,7-TetraCN	1,2,3,5,7-PentaCN	1,2,3,5,6,7-HexaCN
1,2,3,4,5,6,7-HeptaCN	OctaCN		

Table S2. Toxic equivalency factors (TEFs) of PCDD/Fs, PCBs, and PCNs

compound	TEF	compound	TEF
2378-TCDF	0.1	CB-77	0.0001[a]
12378-PeCDF	0.03	CB-81	0.0003[a]
23478-PeCDF	0.3	CN-1	0.000017[a]
234678-HxCDF	0.1	CN-10	0.000027[a]
123478-HxCDF	0.1	CN-2	0.000018[a]
123789-HxCDF	0.1	CN-38/40	0.000008[b]
123678-HxCDF	0.1	CN-4	0.00000002[a]
1234678-HpCDF	0.01	CN-42	0.0000075[a]
1234789-HpCDF	0.01	CN-45/36	0.00000041[a]
OCDF	0.0003	CN-48/35	0.000021[a]
2378-TCDD	1	CN-50	0.000068[a]
12378-PeCDD	1	CN-52/60	0.0000042[a]
123478-HxCDD	0.1	CN-53/55	0.0000018[a]
123678-HxCDD	0.1	CN-54	0.00017[a]
123789-HxCDD	0.1	CN-56	0.000046[a]
1234678-HpCDD	0.01	CN-57	0.0000037[a]
OCDD	0.0003	CN-61	0.00000042[a]

CB-105	0.00003	CN-63	0.002[a]
CB-114	0.00003	CN-64/68	0.00002[a]
CB-118	0.00003	CN-65/70	0.0011[a]
CB-123	0.00003	CN-66/67	0.0039[a]
CB-126	0.1	CN-69	0.002[a]
CB-156	0.00003	CN-71/72	0.0000035[b]
CB-157	0.00003	CN-73	0.003[b]
CB-167	0.00003	CN-74	0.0000041[a]
CB-169	0.03	CN-75	0.00001[a]
CB-189	0.00003		

[a]From a previous study [2]

[b]From a previous study [3]

[c]The CN-65/70 peaks could not be separated by GC-MS/MS, and the concentrations was halved when calculating the TEQ concentrations.

Risk assessment equations

Noncarcinogenic

$$\text{no-CR}_{\text{der}} = \frac{\text{C}_\text{S} \times \text{EF} \times SA \times AF \times ABS \times 10^{-6}}{RfD_O \times AT \times BW \times GIABS}$$

$$\text{no-CR}_{\text{ing}} = \frac{\text{C}_S \times \text{EF} \times ED \times RBA \times IRS \times 10^{-6}}{RfD_O \times AT \times BW}$$

$$\text{no-}CR_{\text{inh}} = \frac{\text{C}_\text{S} \times \text{EF} \times ED \times ET}{RfC \times AT} \times (\frac{1}{VF} + \frac{1}{PEF})$$

$$\text{no-}CR = \text{no-CR}_{\text{der}} + \text{no-CR}_{\text{ing}} + \text{no-}CR_{\text{inh}}$$

Carcinogenic

$$CR_{der} = \frac{C_S \times DFS \times ABS \times 10^{-6}}{AT} \times \frac{SF_O}{GIABS}$$

$$DFS_{resident} = \frac{EF \times ED_C \times SA_C \times AF_C}{BW_C} + \frac{EF \times (EDa - EDc) \times SA_a \times AF_a}{BW_a}$$

$$CR_{ing} = \frac{C_S \times SF_O \times RBA \times IFS \times 10^{-6}}{AT}$$

$$IFS_{resident} = \frac{EF \times ED_C \times IRS_C}{BW_C} + \frac{EF \times (ED_a - ED_c) \times IRS_a}{BW_a}$$

$$CRinh = \frac{C_S \times EF \times ED \times ET \times IUR \times 1000\ \mu g/mg}{AT} \times (\frac{1}{VF} + \frac{1}{PEF})$$

$$CR = CRder + CRing + CRinh$$

Where:
no-CR=Total non-carcinogenic risks
CR= Total carcinogenic risk
no-CRing= Total non-carcinogenic risks through ingestion
no-CRder= Total non-carcinogenic risks through dermal contact
no-CRinh= Total non-carcinogenic risks through inhalation
CRing=Total carcinogenic risk through ingestion
CRder=Total carcinogenic risk through dermal contact
CRinh=Total carcinogenic risk through inhalation
GIABS=1
10^{-6} =correction factor (mg kg-1)
-Other values can be found in Table S3 and Table S4

Table S3. Calculation parameters used in the health risk assessment [4–5]

parameter		units	residential	
IFS	soil ingestion factor adjusted	mg kg^{-1}	36750	
DFS	soil dermal contact factor adjusted	mg kg^{-1}	103390	
AT_{-CR}	averaging time	d	25550	
			child	adult
EF	exposure frequency	d y^{-1}	350	350
ED	exposure duration	y	6	26
ET	exposure time	hh^{-1}	24/24	24/24
$AT_{-no\text{-}CR}$	averaging time	d	2190	9490
BW	body weight	kg	15	80
IRS	soil ingestion rate	mg d^{-1}	200	100
SA	surface area	cm^2 d^{-1}	2373	6032

AF	adherence factor soil to skin	mg cm^2	0.2	0.07

Table S4. Compound specific parameters used in the health risk assessment [4]

parameter		units	2,3,7,8-TCDD
RfDo	oral reference dose	mg kg^{-1}d^{-1}	7.00×10^{-10}
RfC	reference concentration	mg m^{-3}	4.00×10^{-8}
ABS	absorption fraction	-	0.03
VF	volatilization factor	m^3 kg^{-1}	1.96×10^6
PEF	particulate emission factor	m^3 kg^{-1}	1.36×10^9
Sfo	oral slope factor	(mg kg^{-1} d^{-1})$^{-1}$	1.30×10^5
IUR	inhalation unit risk	(ug/m^3)$^{-1}$	38
RBA	Relative Bioavailable	-	1

Table S5. Pearson correlation coefficients for the relationships between the soil and fly ash samples of PCN, PCB and PCDD/Fs homologs

PCN					
	0-500m	500-1000m	1000-1500m	1500-2000m	2000-2500m
500-1000m	0.914**				
1000-1500m	0.934**	0.933**			
1500-2000m	0.730*	0.883**	0.759*		
2000-2500m	0.814*	0.969**	0.830*	0.948**	
Fly ash	0.994**	0.873**	0.926**	0.655	0.750*
PCB					
	0-500m	500-1000m	1000-1500m	1500-2000m	2000-2500m
500-1000m	0.978**				
1000-1500m	0.989**	0.998**			
1500-2000m	0.980**	0.982**	0.986**		
2000-2500m	0.898*	0.883*	0.886*	0.811	
Fly ash	0.958**	0.925**	0.940**	0.976**	0.751
PCDD/Fs					

	0-500m	500-1000m	1000-1500m	1500-2000m	2000-2500m
500-1000m	0.987**				
1000-1500m	0.988**	0.978**			
1500-2000m	0.976**	0.990**	0.965**		
2000-2500m	0.914**	0.956**	0.883**	0.933**	
Fly ash	0.046	0.127	0.001	0.078	0.282

* significantly correlated at P<0.05 (2-tailed);** was significantly correlated at P<0.01 (2-tailed).

Table S6. Carcinogenic risks (CR) and non-carcinogenic risks (no-CR) near by the cement kilns co-processing municipal waste

Samples	CR_{child}	no-CR_{child}	CR_{adult}	no-CR_{adult}
A1	6.39×10^{-8}	6.12×10^{-3}	6.56×10^{-8}	6.07×10^{-4}
A2	3.12×10^{-8}	2.99×10^{-3}	3.20×10^{-8}	2.97×10^{-4}
A3	3.62×10^{-8}	3.46×10^{-3}	3.71×10^{-8}	3.43×10^{-4}
A4	2.95×10^{-8}	2.83×10^{-3}	3.03×10^{-8}	2.80×10^{-4}
A5	3.65×10^{-8}	3.50×10^{-3}	3.75×10^{-8}	3.47×10^{-4}
A6	2.85×10^{-8}	2.73×10^{-3}	2.92×10^{-8}	2.70×10^{-4}
A7	3.04×10^{-8}	2.91×10^{-3}	3.12×10^{-8}	2.89×10^{-4}
A8	3.51×10^{-8}	3.36×10^{-3}	3.60×10^{-8}	3.34×10^{-4}
A9	2.67×10^{-8}	2.55×10^{-3}	2.74×10^{-8}	2.53×10^{-4}
A10	2.88×10^{-8}	2.76×10^{-3}	2.96×10^{-8}	2.74×10^{-4}
A11	6.48×10^{-8}	6.21×10^{-3}	6.65×10^{-8}	6.16×10^{-4}
A12	4.79×10^{-8}	4.59×10^{-3}	4.92×10^{-8}	4.55×10^{-4}
A13	8.93×10^{-8}	8.55×10^{-3}	9.16×10^{-8}	8.48×10^{-4}
A14	1.65×10^{-8}	1.58×10^{-3}	1.70×10^{-8}	1.57×10^{-4}
A15	4.30×10^{-8}	4.12×10^{-3}	4.41×10^{-8}	4.08×10^{-4}
A16	2.92×10^{-8}	2.80×10^{-3}	3.00×10^{-8}	2.77×10^{-4}
A17	4.01×10^{-8}	1.57×10^{-3}	4.12×10^{-8}	3.81×10^{-4}

Table S7. Proportions of carcinogenic risk (CR) and non-carcinogenic risks (no-CR) in different pathways (%)

	ingestion	dermal	inhalation
CR_{child}	91.5	7.72	0.78

CR_{adult}	89.18	7.53	3.3
no-CR_{child}	93.3	6.64	0.06
no-CR_{adult}	89.04	8.04	2.92

Reference

1. Günter B.; Ulrich V. Experimental determination of the effect of mountain-valley breeze circulation on air pollution in the vicinity of Freiburg. Atmos. Environ. **1999,** 33, 4019–4027.

2. Falandysz J, Fernandes A, Gregoraszczuk E, *et al.* The toxicological effects of halogenated naphthalenes: a review of aryl hydrocarbon receptor-mediated (dioxin-like) relative potency factors[J]. J. Environ. Sci. Health, Part C **2014,** 32 (3): 239.

3. Li L, Sun S, Dong S, *et al.* Polychlorinated naphthalene concentrations and profiles in cheese and butter, and comparisons with polychlorinated dibenzo--dioxin, polychlorinated dibenzofuran and polychlorinated biphenyl concentrations[J]. Int. J. Environ. Anal. Chem. **2015,** 95 (3): 203-216.

4.USEPA. Risk-assessment Guidance for Superfund. Volume 1. Human Health Evaluation Manual (Part B, Development of Risk-based Preliminary Remediation Goals). 1991. Available online: https://nepis.epa.gov/Exe/ZyPURL.cgi?Dockey=9100UGUU.txt.(accessed on December 1991)

5.USEPA. Regional Screening Levels (RSLs) — Generic Tables. 2017. Available online: https://www.epa.gov/risk/regional- screening-levels-rsls-generic-tables-june-2017 (accessed on June 2017).
